# Pridinol treatment in cervical dystonia: improvement of non-motor symptoms

**DOI:** 10.1080/07853890.2026.2715209

**Published:** 2026-08-20

**Authors:** Jos Steffen Becktepe, Alexander Baumann, Dana-Kristin Brinker, Carl Alexander Gless, Inken Toedt, Kirsten E. Zeuner

**Affiliations:** ^a^ Department of Neurology, Universitätsklinikum Schleswig-Holstein, Kiel, Germany; ^b^ August-Bier-Klinik, Specialist clinic for neurology and neurorehabilitation, Bad Malente-Gremsmühlen, Germany; ^c^ Wiegmann-Klinik, Clinic of Psychosomatic Medicine and Psychotherapy, Clinic of the German Red Cross Westend, Berlin, Germany; ^d^ Department of Radiology and Neuroradiology, Universitätsklinikum Schleswig-Holstein, Kiel, Germany; ^e^ Institute of Sexual Medicine & Forensic Psychiatry and Psychotherapy, Universitätsklinikum Schleswig-Holstein, Kiel, Germany

**Keywords:** Cervical dystonia, pridinol, pain, depression, botulinum toxin

## Abstract

**Background**: Pridinol mesylate is an anticholinergic medication licensed for the treatment of cervical dystonia (CD) in Germany. We intended to assess the drug’s efficacy on motor and non-motor symptoms in CD-patients.

**Methods:**

This non-interventional pilot study enrolled 20 CD-patients. Pridinol mesylate was prescribed additionally to the standard botulinum toxin (BoNT)-treatment at the initial visit (V1). Follow-up visits were performed after 4 (V2) and after 12 weeks (V3). Motor and non-motor symptoms were assessed using the Toronto Western Spasmodic Torticollis Rating Scale (TWSTRS), the Dystonia Non-Motor Symptoms Questionnaire (DNMSQuest), the Beck Depression Inventory (BDI-II), and the EQ-5D-5L Quality of Life assessment.

**Results:**

The TWSTRS part I motor score and BDI-II significantly improved from V1 to V3. The TWSTRS part III (pain) showed some effect between V1 and V2 and remained unchanged until V3. There was a significant negative correlation between the visual analog scale of the EQ-5D-5L and TWSTRS and a trend towards a positive correlation between TWSTRS part III and BDI-II.

**Discussion:**

Our findings suggest, that pridinol mesylate has some additional clinical effect on motor symptoms and pain when added to BoNT-treatment.

## Introduction

Idiopathic cervical dystonia (CD) is one of the most common forms of focal dystonia to occur in adults, with an estimated prevalence of 23–130 cases per million people [1,2]. CD has a great impact on the activities of daily living (ADL) and quality of life [3,4]. In a Finnish population study, 39% of CD patients had retired because of CD at a median age of 48 years [5].

Clinically, CD is characterized by sustained or intermittent contractions of the cervical muscles that generate abnormal movements and postures [2]. Patients with CD often present with additional non-motor symptoms [6–10]. Pain is the most frequent one, occurring in 55% to 89% of CD patients [11–14], and has great impact on quality of life. Another very important non-motor characteristic is depression, which is associated with a higher prevalence of suicidal behavior [15,16].

Chemodenervation with botulinum toxin (BoNT) is the most effective, worldwide standard treatment of choice for CD [7]. It does improve quality of life and reduces pain in 71% of patients [17]. However, BoNT should not be injected more frequently than every 10–12 weeks to avoid immune resistance and minimize side effects [18].

In case BoNT is not effective, alternative drugs are used. Most of them include anticholinergics, such as trihexyphenidyl, benztropine, biperiden, ethopropazine, orphenadrine, and procyclidine [19,20]. They are considered to block muscarinic acetylcholine receptors in the basal ganglia [19]. In Germany, the anticholinergic drug most frequently used is trihexyphenidyl. However, trihexyphenidyl has only been evaluated in one prospective, double-blind trial in patients with an average age of 19 years who tolerated high doses of up to 30 mg, while older patients are less likely to tolerate its many side effects, including memory loss, confusion, agitation, depression, dry mouth, constipation, urinary retention, blurred vision or worsening of narrow-angle glaucoma [19]. Based on limited evidence, BoNT injections provide more objective and subjective benefit than trihexyphenidyl to patients with cervical dystonia [20,21].

Clonazepam is an effective oral treatment option. However, the side-effect profile of clonazepam, including sedation, impaired mentation and coordination, depression, and the risk of tachyphylaxis and dependence, prevents its routine use [19].

In conclusion, BoNT has good efficacy in CD, but the effect of BoNT diminishes after 8–12 weeks with recurrent motor and non-motor symptoms. The most commonly used oral medications (trihexyphenidyl and clonazepam) improve motor and non-motor symptoms but also cause a number of side effects. Pridinol mesylate 4 mg (Myopridin ^®^ 3 mg, Strathmann, Germany) is another anticholinergic drug, which is licensed in several European countries including Germany, United Kingdom, Poland, and Spain for the treatment of muscle spasms and torticollis (without specifying what type of torticollis it is, i.e. muscular or cervical dystonia). Pridinol mesylate reaches its maximum plasma concentration within 1 h after oral administration and is renally eliminated [22]. In a recent meta-analysis of placebo-controlled studies in adult patients with mild to moderate muscle pain, pridinol showed a comparable safety profile, but significantly better analgesic effects and improvements in muscle stiffness, tenderness, and movement limitations compared with placebo [23]. To date, there have been no clinical trials investigating the effect of pridinol on the motor and non-motor symptoms experienced by patients with cervical dystonia. Pridinol may therefore offer a better tolerated alternative to the established drugs used to treat CD.

In our non-interventional study, we aimed to evaluate how improvements in motor symptoms, as measured by the Toronto Western Spasmodic Torticollis Rating Scale (TWSTRS) part I, affect non-motor symptoms in patients with CD who receive regular BoNT plus pridinol mesylate treatment.

## Methods

### Definition of the study cohort

A total of 20 patients were recruited for this non-interventional study from the BoNT therapy outpatient clinics at Kiel University Hospital between October 2021 and August 2023. The trial was approved by the local ethics committees of the University of Kiel (D 527/21). All patients gave a written informed consent to participate. The study was registered in the public German registry for clinical studies (DRKS number 00025427) before the time of first patient enrollment.

To be eligible for the study, patients had to fulfill the following criteria:

*Inclusion criteria:*

- Cervical dystonia with a diagnosis established > 2 years ago
- Last BoNT injection 12 weeks before study inclusion
- Medical indication for pridinol.

*Exclusion criteria:*

- Generalized dystonia
- Severe depression, psychosis, dementia
- Drug or alcohol abuse
- Glaucoma, prostate hypertrophy, urinary retention, gastrointestinal obstruction, cardiac arrhythmia, pregnancy and the use of benzodiazepines or any anticholinergics within three months prior to inclusion.

### Description of the clinical questionnaires

#### Toronto Western spasmodic Torticollis rating scale (TWSTRS)

The TWSTRS is a validated standard rating scale for treatment interventions in CD. It includes a motor (severity) part I, a disability part II and part III for pain. The TWSTRS-severity estimates the clinical presentation on a score between 0 to 30 points. The TWSTRS-Disability is a seven-item scale that ranges between 0 and 30 points that assesses the impact of CD on patients’ ability to perform daily activities. The TWSTRS-Pain consists of a severity score for the patient’s usual, worst, and best pain and its duration with a score between 0 and 20 points [24].

#### Dystonia non-motor symptoms questionnaire (DNMSQuest)

This fourteen-question validated questionnaire provides an overview of various non-motor symptoms. The scale covers seven different domains (sleep, autonomic symptoms, fatigue, emotional well-being, stigma, activities of daily living, sensory symptoms). The DNMSQuest ranges between 0 and 14 points [25].

#### Beck depression inventory (BDI-II)

The BDI-II is a self-assessment scale comprising 21 questions on depressive symptoms, each with four possible answers of different symptom severity, which are to be rated in terms of their presence in the last two weeks. Die BDI-cumulative values are between 0 and 63 points [26].

#### Quality of life (EQ-5D-5L)

The EQ-5D-5L is a validated, standardized measure of health-related quality of life. The following dimensions are examined: mobility, self-care, usual activities, pain/depression, anxiety/depression. On a five-level scale level 1 indicates no problems and level 5 represents extreme difficulties. The resulting 5-digit number can be converted into a point value (EQ5D-5L Index) using a special algorithm that is not publicly accessible. Additionally, the EQ VAS records the patient’s self-rated health on a vertical visual analogue scale of 0 to 100 points, where the endpoints are labelled ‘The best health you can imagine’ and ‘The worst health you can imagine’ [27].

### Treatment with BoNT and pridinol mesylate

The following BoNT preparations were used: Onabotulinumtoxin A (Botox^®^, AbbVie company, USA, in 7 patients), Abobotulinumtoxin A (Dysport^®^, Ipsen Pharma, France, in 4 patients) and Incobotulinumtoxin A (Xeomin^®^, Merz Pharmaceuticals, Germany, in 8 patients). EMG guidance was not routinely used for BoNT injections, ultrasound guidance was employed when injecting deep cervical muscles (e.g. obliquus capitis inferior muscle). One patient was currently not treated with BoNT. If a patient was not adequately treated with BoNT, we offered them several options to improve efficacy:

1. Increase the dose of BoNT
2. Prescribe additional physiotherapy
3. Prescribe an anticholinergic medication (trihexyphenidyl or pridinol mesylate)
4. Prescribe clonazepam.

In case the patient agreed to pridinol mesylate, we prescribed this medication, informed them about our non-interventional trial and asked them to participate.

As part of our routine clinical procedure with patients who are not satisfied with their current treatment, we performed three visits for evaluation and applied all scales at each visit:

Visit 1 (V1) was the baseline visit. Each patient received the regular BoNT-treatment and was prescribed Myopridin 3 mg tablets (Strathmann, Germany). Each Myopridin tablet contains 3 mg of pridinol, as well as mesylate salt. This results in a total of 4 mg of pridinol mesylate per tablet. They were advised to start with ½ a tablet three times a day from the next day after V1 on and to increase the dose to one tablet three times a day after one week. The medication was therefore started within one day after v1.

Visit 2 (V2) was the follow up visit four weeks after the injection and the beginning of the pridinol medication.

**Figure 1.**
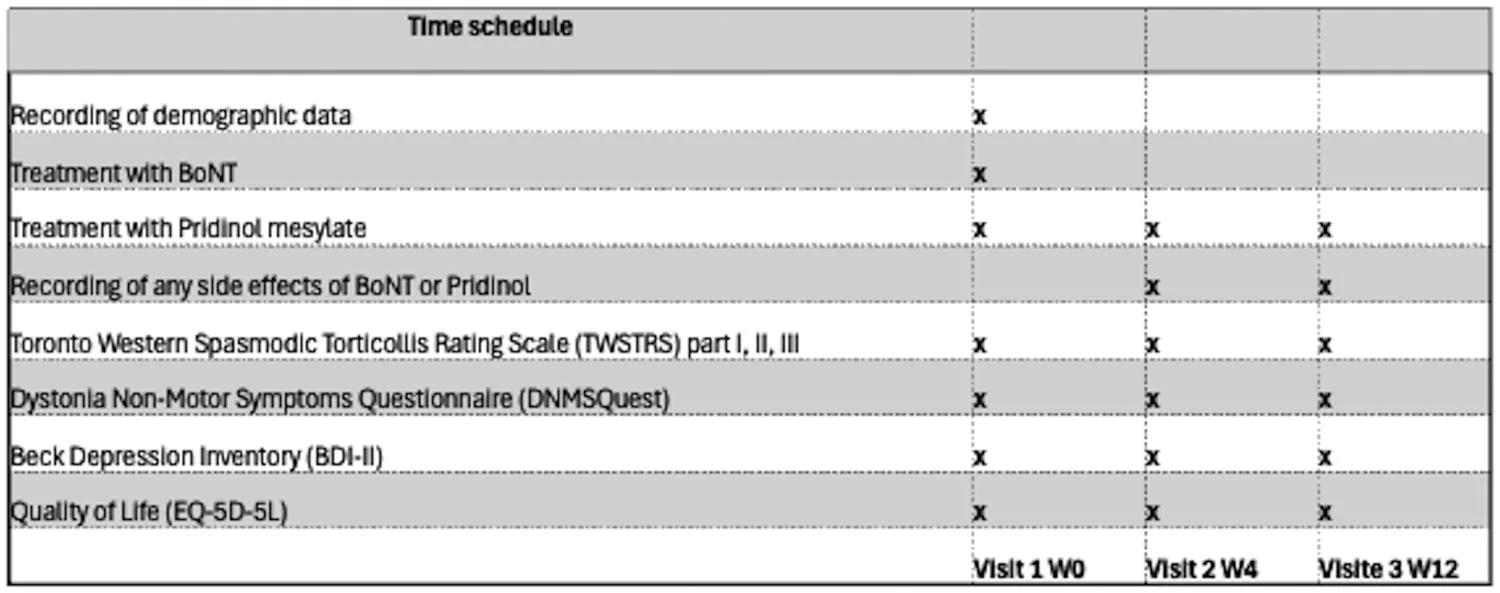
Time schedule of the non-interventional study.

Visit 3 (V3) was the second injection visit, 12 weeks after the last BoNT injection (and therefore with diminished effect of BoNT) but with ongoing pridinol treatment (Figure 1). Patients were given the choice of continuing with pridinol or stopping the medication if it was not effective.

### Statistical analysis

Due to the small sample size, nonparametric tests were preferred. Friedman test was used for one-way repeated measures analysis of variance and Conover′s test of multiple comparisons with Holm correction was applied for post-hoc testing. The correlation of changes between different clinical motor- and non-motor scores between V1 and V3 were analyzed in a Spearman rank correlation. A bootstrapped linear regression model with 5000 replicates was calculated to examine the influence of change in pain (TWSTRS part III) on depression (BDI-II). Only complete datasets from patients who continued to take Pridinol medication until V3 were included in the repeated measures and regression analyses. The statistical analysis was performed with JASP (Version 0.19.3).

## Results

### Demographic and descriptive data of patients

The study cohort included 20 patients (7 females) with idiopathic cervical dystonia, a mean age of 56.5 (SD ± 12.0) years and a mean disease duration of 23.2 (SD ± 14.1) years (Table 1). Twelve out of twenty patients attended all three visits (Supplementary Figure 1).

**Table 1.** Demographic data, patients attended (1) or not attended (0) visits V1, V2, V3, pridinol mesylate (Myopridin ^®^) dosages at V2 and V3, side effects of all patients.

Pat. No.	V1	V2	V3	Tremor No ≙ 0 Yes ≙ 1	Sex ♀ ≙ 1 ♂ ≙ 0	Age	Disease onset	Myopridin^®^ Dosage per day at V2-V3	Side effects
NMS-CD_01	1	0	0	0	1	46	2008	0	No Myopridin^®^ intake
NMS-CD_02	1	1	1	0	1	60	2007	4.5 mg–6 mg	Dizziness, dry mouth
NMS-CD_03	1	1	1	1	1	46	2002	4.5 mg–9 mg	Tremor of the hands and voice, impaired coordination, diarrhea, nausea, dry mouth, tiredness, polyuria, transient cervical pain
NMS-CD_04	1	1	1	1	1	53	1980	3 mg–4.5 mg	Restlessness, dry mouth, tiredness, transient word-finding disorder, reduced appetite
NMS-CD_05	1	1	1	0	0	45	2019	9 mg	Impaired concentration and accommodation, tiredness
NMS-CD_06	1	1	1	1	1	54	2010	9 mg	Tiredness
NMS-CD_07	1	1	0	0	1	60	2014	9 mg	Tiredness, dry mouth
NMS-CD_08	1	1	0	0	1	51	2010	9 mg	Dry mouth, increased appetite, stopped medication after 8 weeks
NMS-CD_09	1	1	0	1	1	78	2000	4.5 mg–9 mg.	No side effects with 3 × 0,5 mg, with 3 × 3 mg dizziness, concentration deficits, coordination disorder, visual disturbances, abdominal pain; stopped after 2 weeks
NMS-CD_10	1	1	1	0	1	66	1970	9 mg	none
NMS-CD_11	1	1	1	0	0	50	1998	3 mg	tiredness, thumb tremor, discontinued after 8 weeks
NMS-CD_12	1	1	1	0	1	48	2006	9 mg	Dry mouth, discontinued after 3 months
NMS-CD_13	1	1	0	0	1	60	1990	4.5 mg	Dry mouth, nausea; discontinued after 3 weeks
NMS-CD_14	1	1	1	1	1	69	1985	9 mg	Tremor of the left hand, dry mouth, tiredness, impaired coordination of the legs, short-term memory impairment, dry mouth
NMS-CD_15	1	1	1	1	0	40	2006	3 mg–9 mg	Tiredness
NMS-CD_16	1	0	0	1	1	82	1970	0	Not known; patient has not presented again.
NMS-CD_17	1	1	1	0	1	56	2010	3 mg–6 mg	none
NMS-CD_18	1	0	0	0	1	37	2007	2 × 1.5 mg	Discontinued after two weeks due to fatigue
NMS-CD_20	1	1	1	0	1	68	1996	6 mg at V2, reduced to 4.5 mg at V3	Transient speech disorder Transient concentration deficit, tiredness, sleep impaired
NMS-CD_21	1	1	1	1	1	61	1998	9 mg	Dry mouth

Patients who used pridinol mesylate but did not attend all visits did so for the following reasons:

No reason given (*n* = 2), discontinued due to missing effects (*n* = 5), not present for examination (*n* = 1). Eight patients were taking less than the recommended 9 mg Myopridin^®^ at V2. Three patients had discontinued pridinol mesylate prematurely. At the time of V3, eight patients were taking the recommended dosage of 9 mg daily, four patients remained on a reduced dosage, and two patients had discontinued Myopridin^®^ four weeks earlier because of tiredness. The most common adverse reactions were dry mouth (*n* = 9), tiredness (*n* = 9), impaired concentration (*n* = 3), dizziness (*n* = 3), or impaired coordination (*n* = 3).

### Toronto Western spasmodic Torticollis rating scale (TWSTRS)

There was a significant improvement of the TWSTRS total score after BoNT treatment and the start of pridinol mesylate medication as shown by the TWSTRS total score (Friedmann test: χ^2^ = 8.167; *p* = 0.017; *n* = 12).

**Figure 2.**
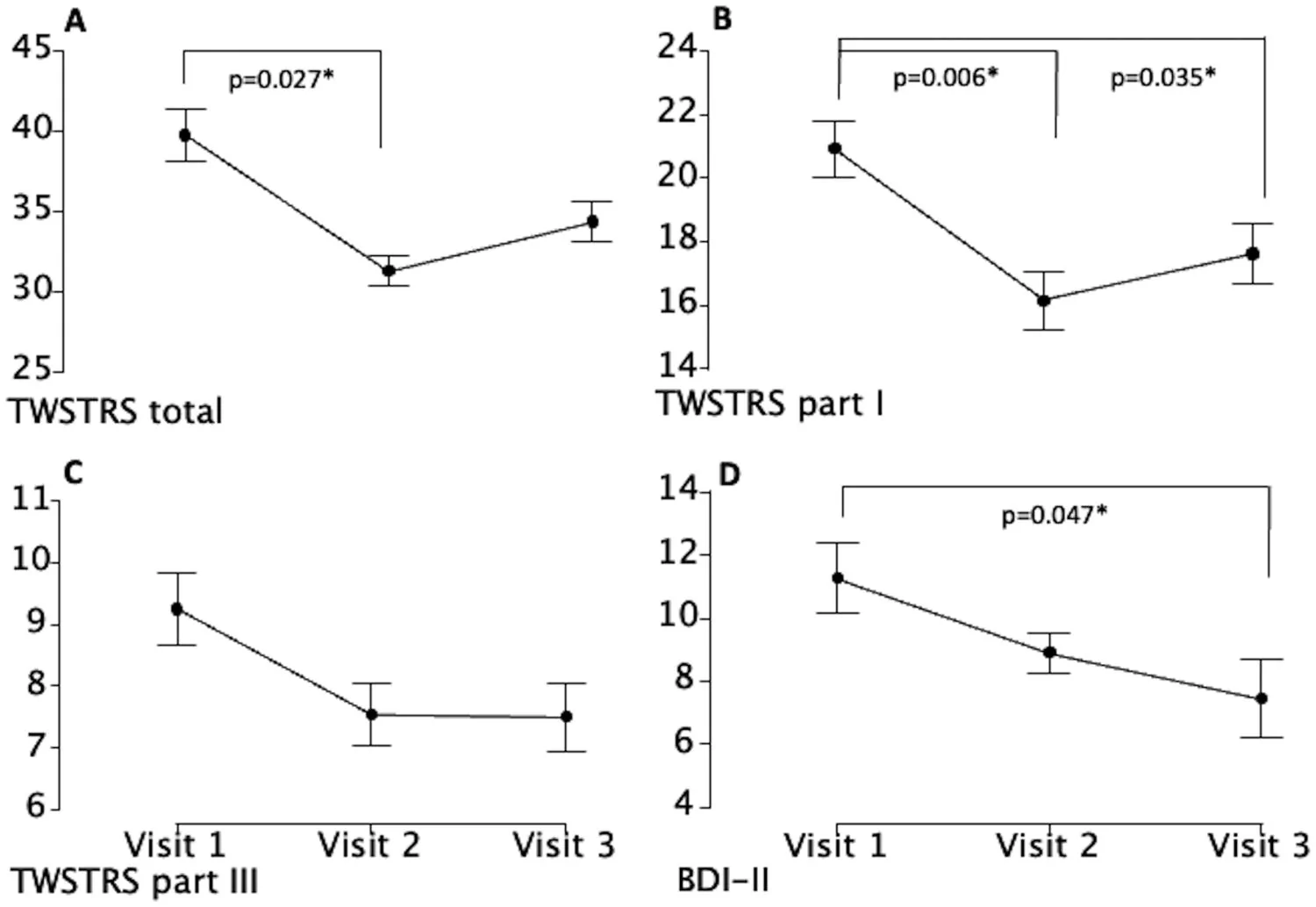
Mean values and standard error of clinical scales at visits 1–3; (A) TWSTRS total score was significantly lower at visit 2 (p_holm_ = 0.027) compared to visit 1, but there was no significant difference between visit 1 and visit 3; (B) TWSTRS Part I was significantly reduced at visit 2 (p_holm_ = 0.006) and visit 3 (p_holm_ = 0.035); (C) TWSTRS part III exhibited no significant differences; (D) The BDI-II mean score continuously decreased from visit 1 to visit 3 and remained significantly reduced at visit 3 (p_holm_ = 0.047).

Conover′s post-hoc analysis indicated that the main effect of the reduction in TWSTRS total score was a significant therapeutic effect between V1 and V2 (p_holm_=0.027), but not between V1 and V3 (p_holm_=0.334) (Figure 2).

The analysis of the individual TWSTRS subscales revealed a reduction in part I (χ^2^ = 13.042; *p* = 0.001, *n* = 12). The post-hoc t-test demonstrated a significant difference not only between V1 and V2 (p_holm_=0.006), but also between V1 and V3 (p_holm_=0.035) (Figure 2).

There was no change in the disability score after V1 in the TWSTRS part II (χ^2^ = 2.711; *p* = 0.258) (Supplementary Figure 2).

TWSTRS Part III (Pain) exhibited no significant differences (χ^2^ = 3.522; *p* = 0.172). It is noteworthy that the pain score remained almost unchanged between V2 and V3 (Figure 2).

### Non-motor scores: dystonia non-motor symptoms questionnaire (DNMSQuest), beck depression inventory (BDI-II), quality of life (EQ 5D-5L)

There were no significant effects on the non-motor questionnaire (χ^2^=0.341; *p* = 0.843; *n* = 12) and quality of life (χ^2^=2.579; *p* = 0.275) (Supplementary Figure 2), but a trend on the BDI-II (χ^2^ = 5.692; *p* = 0.058). Post hoc analysis revealed a significant difference between V1 and V3 (p_holm_=0.047) (Figure 2).

### Relationship between changes in motor and non-motor scores

Spearman’s rank correlation showed no significant correlations between the changes in clinical motor- and non-motor scores between V1 and V3 according to the change of total TWSTRS, part I and part III with the BDI-II and the DNMSQuest. However, the change in total TWSTRS was negatively correlated with the visual analogue scale of the EQ-5D-5L (*ρ* = 0.612; *p* = 0.046; Table 2), meaning patient’s self-rated health improved as motor symptoms recovered. There was a trend for a positive correlation between the TWSTRS part III and the BDI-II (*ρ* = 0. 0.529; *p* = 0.094; Table 2).

**Table 2.** Results of non-parametric spearman rank correlation analysis.

Variable		Delta BDI	Delta EQ5D Index	Delta EQVAS	Delta DNMSQuest	Delta TWSTRS1	Delta TWSTRS 3	Delta TWSTRS
Delta BDI	Spear-man’s rho	–						
	*p*-value	–						
Delta EQ5D Index	Spear-man’s rho	−0.215	–					
	*p*-value	0.526	–					
Delta EQVAS	Spear-man’s rho	−0.488	0.469	–				
	*p*-value	0.127	0.145	–				
Delta DNMS Quest	Spear-man’s rho	0.430	−0.019	−0.481	–			
	*p*-value	0.187	0,.956	0.134	–			
Delta TWSTRS 1	Spear-man’s rho	0.106	−0.464	−0.533	0.009	–		
	*p*-value	0.756	0.150	0.091	0.978	–		
Delta TWSTRS 3	Spear-man’s rho	0.529	−0.014	−0.395	0.264	−0.102	–	
	*p*-value	0.094	0.967	0.229	0.407	0.753	–	
Delta TWSTRS	Spear-man’s rho	0.345	−0.410	−0.612	0.481	0.642	0.536	–
	*p*-value	0.299	0.210	**0.046**	0.114	**0.024**	0.073	–

A bootstrapped linear regression model with change in pain (delta TWSTRS part III) as independent variable and change in depression (delta BDI-II) as dependent variable was not significant (adjusted R^2^ = 0.159; *p* = 0.123). However, the coefficient estimate for delta TWSTRS part III as intercept showed a non-significant trend (*p* = 0.072; Suppl. Table 1).

## Discussion

BoNT is known to have an excellent effect on motor and non-motor symptoms. However, the effect on motor and non-motor symptoms wanes after 8–12 weeks. Additional medications may facilitate or prolong the effect of BoNT. One possible medication is pridinol mesylate, which is approved for cervical dystonia in several European countries. The aim of our non-interventional study was to determine the relationship between improved motor and non-motor symptoms in patients with CD receiving regular BoNT plus pridinol mesylate.

We obtained the following major results:

1. As expected, the total TWSTRS score decreased significantly after treatment with BoNT plus pridinol mesylate. The score dropped by 8.5 points from mean 39.8 to 31.3 points between V1 and V2 but this effect waned between V1 and V3.
2. TWSTRS part I significantly improved between V1 and V2 (*p* = 0.006) and this improvement was sustained until V3 (*p* = 0.035).
3. TWSTRS part III showed a trend effect (*p* = 0.073) on pain after BoNT and pridinol mesylate treatment between V1 and V2 from 9.5 to 7.8 points. The score remained lower through V3 at 7.9 points compared to V1.
4. Depression as measured by the BDI-II decreased continuously from 11.3 points at V1 to 7.5 points at V3. The therapeutic effect was significant between V1 and V3 (*p* = 0.027).
5. The total TWSTRS was negatively correlated with the EQ-5D-5L visual analog scale, indicating that quality of life increased with improvement in motor symptoms, pain, and disability.
6. There was a trend for a positive correlation between pain (TWSTRS part III) and the BDI-II, indicating that a reduction in pain has a positive effect on depression. This finding is underlined be the results of a bootstrapped linear regression model.

The significant improvement of 27.5% (8 points) in the TWSTRS total score four weeks after treatment with BoNT and pridinol mesylate is not surprising and has been reported previously. In a systematic review, the mean reduction of 8.09 points in the TWSTRS total score four weeks after injection corresponded to a mean improvement of 18.4% from baseline [20]. The improvement in the TWSTRS total score at V2 appears to be mainly due to the significant reduction in the TWSTRS part I score, whereas TWSTRS parts II and III show a trend towards improvement but no significant improvement, possibly due to the small sample size. However, the improvement of TWSTRS total score waned until V3. Of note, the significant improvement of TWSTRS part I was sustained until V3. This is interesting, because the effect of BoNT usually wanes after 8–12 weeks. Therefore, it is conceivable that pridinol mesylate had an additional effect on motor symptoms and contributed to the lower TWSTRS part I at three months. BoNT should be injected at intervals between 10 and 12 weeks [17]. Whereas 71.6% of patients are very satisfied 3.6 weeks after their injection, 45.6% of patients would prefer injection cycles of <10 weeks [17]. Pridinol mesylate may be a viable option to increase the interval between repeated injections for those patients.

Patients with CD experience pain [28], which affects their emotional well-being [3,29]. BoNT significantly reduces pain [17], although in 84% of patients, pain is the first non-motor symptom to return, even before motor symptoms are detected [30]. The recurrence of pre-existing symptoms occurred after 73.6 days, or 2.4 months [30]. In our study, the TWSTRS Part II score for disability remained unchanged, but the Part III score representing pain declined four weeks after BoNT injection and initiation of pridinol mesylate. This decrease was not quite significant, probably due to the small number of patients. However, it is noteworthy that the pain score remained at the same level even three months after the last BoNT treatment, which cannot be explained by the BoNT effect alone. Pridinol mesylate improves pain in patients with spondylarthritis and thus facilitates rehabilitation [31]. It also reduces the pressure pain threshold in experimental temporomandibular joint pain [32]. Although not significant, it is conceivable that the low pain score at V3 is caused by the effect of pridinol mesylate treatment.

Besides pain, depression plays a major role in patients with CD [10,33]. Increased pain is associated with worsening of psychiatric symptoms [33]. In our study, depressive symptoms as measured by the BDI-II improved significantly and continuously until V3. Although it remains unclear whether and how pridinol affects depressive symptoms in dystonia patients, one possible mechanism of action could be its tendency to alleviate pain. As described, we found slightly reduced pain at V3 compared to baseline, and pain and depression tended to correlate in our study. Our data suggest that pridinol mesylate causes a reduction in pain and may lead to an improvement of depression. However, with a mean BDI-II score of 11.3 points at baseline, our study sample showed only slight depressive symptoms. It therefore remains unclear whether the effect of pridinol would be more pronounced in more depressed patients.

Although our results suggest a possible positive effect of adding pridinol mesylate to BoNT on motor- and non-motor symptoms in CD, we must acknowledge a relatively high dropout rate due to anticholinergic side effects. Also, recent evidence suggests that cognitive dysfunction may be an intrinsic feature of adult-onset dystonia [34]. Therefore, patients with dystonia may be more likely to experience cognitive adverse effects caused by anticholinergic medications such as pridinol. In summary, pridinol mesylate may only be helpful for a subgroup of patients.

## Conclusion

Our study suggests that pridinol mesylate may have a positive effect on motor and non-motor symptoms in CD, in addition to BoNT. In particular, motor symptoms (TWSTRS part I), pain scores on the TWSTRS III and depression as measured by the BDI-II either remained lower or continuously decreased compared to baseline. This effect can only be partially attributed to the BoNT injections. Therefore, it is conceivable that pridinol mesylate may have some additional effect when added to BoNT treatment.

## Strengths and limitations

This is the first study to investigate pridinol mesylate in addition to standard BoNT treatment and to evaluate its effect on motor and non-motor symptoms and their correlation in patients with CD. The main limitation of the study is its open-label design and lack of a BoNT alone or placebo treated control group. We cannot rule out the possibility, that some of the observed effects are due to the placebo effect or the sustained effects of BoNT, even after 12 weeks. Additionally, the small number of patients who took the medication until the third visit significantly limits the validity of the findings. Only a small number of the patients screened were treated with pridinol mesylate at all, and yet the proportion of treatment discontinuations was relatively high. Therefore, this medication appears to be applicable to only a small proportion of dystonia patients. Further studies with larger patient numbers and a placebo-controlled design are required.

## Supplementary Material

Supplemental Material

Supplementary_table.docx

Suppl_Fig_2.tiff

Suppl_Fig_1.jpg

## Data Availability

The data that support the findings of this study are available on request from the corresponding author, JSB. The data are not publicly available due to restrictions regarding the privacy of research participants.

## References

[1] Defazio G, Abbruzzese G, Livrea P, et al. Epidemiology of primary dystonia. Lancet Neurol. 2004;3(11):673–678. doi:10.1016/S1474-4422(04)00907-X. 15488460

[2] Defazio G, Berardelli A, Hallett M. Do primary adult-onset focal dystonias share aetiological factors? Brain. 2007;130(Pt 5):1183–1193. doi:10.1093/brain/awl355. 17242025

[3] Junker J, Berman BD, Hall J, et al. Quality of life in isolated dystonia: non-motor manifestations matter. J Neurol Neurosurg Psychiatry. 2021;92(6):622–628. doi:10.1136/jnnp-2020-325193. PMC8356023 33563813

[4] Zetterberg L, Aquilonius SM, Lindmark B. Impact of dystonia on quality of life and health in a Swedish population. Acta Neurol Scand. 2009;119(6):376–382. doi:10.1111/j.1600-0404.2008.01111.x. 19016658

[5] Martikainen KK, Luukkaala TH, Marttila RJ. Working capacity and cervical dystonia. Parkinsonism Relat Disord. 2010;16(3):215–217. doi:10.1016/j.parkreldis.2009.07.006. 19660976

[6] Han V, Skorvanek M, Smit M, et al. Prevalence of non‐motor symptoms and their association with quality of life in cervical dystonia. Acta Neurol Scand. 2020;142(6):613–622. doi:10.1111/ane.13304. 32579704

[7] Novaretti N, Cunha ALN, Bezerra TC, et al. The prevalence and correlation of non-motor symptoms in adult patients with idiopathic focal or segmental dystonia. Tremor Other Hyperkinet Mov (N Y). 2019;9(0):596. doi:10.5334/tohm.466. 30783550 PMC6377805

[8] Smit M, Bartels AL, Kuiper A, et al. The frequency and self‐perceived impact on daily life of motor and non‐motor symptoms in cervical dystonia. Mov Disord Clin Pract. 2017;4(5):750–754. doi:10.1002/mdc3.12510. 30363474 PMC6174508

[9] Smit M, Kamphuis AS, Bartels AL, et al. Fatigue, sleep disturbances, and their influence on quality of life in cervical dystonia patients. Mov Disord Clin Pract. 2017;4(4):517–523. doi:10.1002/mdc3.12459. 30363425 PMC6174405

[10] Stamelou M, Edwards MJ, Hallett M, et al. The non-motor syndrome of primary dystonia: clinical and pathophysiological implications. Brain. 2012;135(Pt 6):1668–1681. doi:10.1093/brain/awr224. 21933808 PMC3359748

[11] Marciniec M, Szczepańska-Szerej A, Popek-Marciniec S, et al. Pain incidence in cervical dystonia is determined by the disease phenotype. J Clin Neurosci. 2020;79:133–136. doi:10.1016/j.jocn.2020.07.069. 33070882

[12] Junker J, Hall J, Berman BD, et al. Longitudinal predictors of health-related quality of life in isolated dystonia. J Neurol. 2024;271(2):852–863. doi:10.1007/s00415-023-12022-4. 37839041 PMC10827910

[13] Avenali M, De Icco R, Tinazzi M, et al. Pain in focal dystonias – a focused review to address an important component of the disease. Parkinsonism Relat Disord. 2018;54:17–24. doi:10.1016/j.parkreldis.2018.04.030. 29724604

[14] Maione R, Formica C, Quartarone A, et al. The impact of non-motor symptoms on quality of life in cervical dystonia. J Clin Med. 2023;12(14):4663. doi:10.3390/jcm12144663. 37510780 PMC10380526

[15] Worthley A, Simonyan K. Suicidal ideations and attempts in patients with isolated dystonia. Neurology. 2021;96(11):e1551–e1560. doi:10.1212/WNL.0000000000011596. 33504639 PMC8032380

[16] Zeuner KE, van Gerpen JA. Prevalence of suicidality in focal and generalized dystonia: a critical and unrecognized problem. Hagerstown (MD): Lippincott Williams & Wilkins; 2021. P. 511–512. doi:10.1212/WNL.0000000000011602. 33504643

[17] Contarino MF, Van Den Dool J, Balash Y, et al. Clinical practice: evidence-based recommendations for the treatment of cervical dystonia with botulinum toxin. Front Neurol. 2017;8:35. doi:10.3389/fneur.2017.00035. 28286494 PMC5323428

[18] Bellows S, Jankovic J. Immunogenicity associated with botulinum toxin treatment. Toxins (Basel). 2019;11(9):491. doi:10.3390/toxins11090491. 31454941 PMC6784164

[19] Jinnah HA, Factor SA. Diagnosis & treatment of dystonia. Neurol Clin. 2015;33(1):77–100. doi:10.1016/j.ncl.2014.09.002. 25432724 PMC4248237

[20] Rodrigues FB, Duarte GS, Castelão M, et al. Botulinum toxin type A versus anticholinergics for cervical dystonia. Cochrane Database Syst Rev. 2021;4(4):CD004312. doi:10.1002/14651858.CD004312.pub3. 33852744 PMC8092669

[21] Costa J, Espírito‐Santo CC, Borges AA, et al. Botulinum toxin type A therapy for cervical dystonia. Cochrane Database of Systematic Reviews. 2005;(4):1465-1858. doi:10.1002/14651858.CD003633.pub2. 15674910

[22] Richter M, Donath F, Wedemeyer RS, et al. Pharmacokinetics of oral pridinol: results of a randomized, crossover bioequivalence trial in healthy subjects. Int J Clin Pharmacol Ther. 2021;59(6):471–477. doi:10.5414/CP203900. 33835016 PMC8167741

[23] Ueberall MA, Essner U, Mueller-Schwefe GH. Efficacy and safety/tolerability of pridinol: a meta-analysis of double-blind, randomized, placebo-controlled trials in adult patients with muscle pain. Curr Med Res Opin. 2022;38(7):1141–1151. 35502575 10.1080/03007995.2022.2072089

[24] Jost WH, Hefter H, Stenner A, et al. Rating scales for cervical dystonia: a critical evaluation of tools for outcome assessment of botulinum toxin therapy. J Neural Transm. 2013;120(3):487–496. doi:10.1007/s00702-012-0887-7. 22899277 PMC3575559

[25] Klingelhoefer L, Jost W, Odin P, et al. Dystonia Non-Motor Symptoms Questionnaire (DNMSQuest) for assessment of non-motor symptoms in dystonia: intercultural adaptation in the German language. Nervenarzt. 2020;91(4):337–342. doi:10.1007/s00115-020-00885-1. 32144450

[26] Hautzinger M, Keller F, Kühner C. Beck depressions-inventar (BDI-II). Frankfurt/Main: Harcourt Test Services; 2006.

[27] Group TE. EuroQol-a new facility for the measurement of health-related quality of life. Health Policy (New York). 1990;16(3):199–208. 10.1016/0168-8510(90)90421-9 10109801

[28] Rosales RL, Cuffe L, Regnault B, et al. Pain in cervical dystonia: mechanisms, assessment and treatment. Expert Rev Neurother. 2021;21(10):1125–1134. doi:10.1080/14737175.2021.1984230. 34569398

[29] Klingelhoefer L, Kaiser M, Sauerbier A, et al. Emotional well-being and pain could be a greater determinant of quality of life compared to motor severity in cervical dystonia. J Neural Transm (Vienna). 2021;128(3):305–314. doi:10.1007/s00702-020-02274-z. 33146753 PMC7969693

[30] Comella C, Ferreira JJ, Pain E, et al. Patient perspectives on the therapeutic profile of botulinum neurotoxin type A in cervical dystonia. J Neurol. 2021;268(3):903–912. doi:10.1007/s00415-020-10217-7. 32939574 PMC7914227

[31] Lauricella L, Calabrese N, Scaturro D, et al. Effectiveness rehabilitative therapy and Pridinol Mesylate in low back pain. Front Med. 2025;11:1470996. doi:10.3389/fmed.2024.1470996. PMC11794184 39911667

[32] Svensson P, Wang K, Arendt-Nielsen L. Effect of muscle relaxants on experimental jaw-muscle pain and jaw-stretch reflexes: a double-blind and placebo-controlled trial. Eur J Pain. 2003;7(5):449–456. doi:10.1016/S1090-3801(03)00013-2. 12935797

[33] Berman BD, Junker J, Shelton E, et al. Psychiatric associations of adult-onset focal dystonia phenotypes. J Neurol Neurosurg Psychiatry. 2017;88(7):595–602. doi:10.1136/jnnp-2016-315461. 28438790 PMC5659143

[34] Velucci V, Mascia MM, Avanzino L, et al. Predictors of cognitive performance in idiopathic adult-onset dystonia. J Neural Transm. 2025;133(7):1503–1509. doi:10.1007/s00702-025-03043-6. 41091203

