# Supplementary material for: Pridinol treatment in cervical dystonia: improvement of non-motor symptoms
Source: Ann Med. 2026 Aug 20;58(1):2715209. doi: 10.1080/07853890.2026.2715209 (PMC13501772; doi:10.1080/07853890.2026.2715209)
Supplement: Supplementary file 1 [file IANN_A_2715209_SM0921.docx]

| *Model Summary - delta BDI* | | | | | | | | | |
| --- | --- | --- | --- | --- | --- | --- | --- | --- | --- |
| Model | | R | | R² | | Adjusted R² | | RMSE | |
| M₀ |  | 0.000 |  | 0.000 |  | 0.000 |  | 6.063 |  |
| M₁ |  | 0.493 |  | 0.243 |  | 0.159 |  | 5.559 |  |
|  | | | | | | | | | |
| *Note.*  M₁ includes delta TWSTRS 3 | | | | | | | | | |

| *ANOVA* | | | | | | | | | | | | | |
| --- | --- | --- | --- | --- | --- | --- | --- | --- | --- | --- | --- | --- | --- |
| Model | |  | | Sum of Squares | | df | | Mean Square | | F | | p | |
| M₁ |  | Regression |  | 89.506 |  | 1 |  | 89.506 |  | 2.896 |  | 0.123 |  |
|  |  | Residual |  | 278.130 |  | 9 |  | 30.903 |  |  |  |  |  |
|  |  | Total |  | 367.636 |  | 10 |  |  |  |  |  |  |  |
|  | | | | | | | | | | | | | |
| *Note.*  M₁ includes delta TWSTRS 3 | | | | | | | | | | | | | |
| *Note.*  The intercept model is omitted, as no meaningful information can be shown. | | | | | | | | | | | | | |

| *Coefficients* | | | | | | | | | | | | | |
| --- | --- | --- | --- | --- | --- | --- | --- | --- | --- | --- | --- | --- | --- |
| Model | |  | | Unstandardized | | Standard Error | | Standardized | | t | | p | |
| M₀ |  | (Intercept) |  | 3.818 |  | 1.828 |  |  |  | 2.089 |  | 0.063 |  |
| M₁ |  | (Intercept) |  | 1.916 |  | 2.015 |  |  |  | 0.951 |  | 0.366 |  |
|  |  | delta TWSTRS 3 |  | 1.008 |  | 0.592 |  | 0.493 |  | 1.702 |  | 0.123 |  |
|  | | | | | | | | | | | | | |

| *Bootstrap Coefficients* | | | | | | | | | | | |
| --- | --- | --- | --- | --- | --- | --- | --- | --- | --- | --- | --- |
| Model | |  | | Unstandardized | | Bias | | Standard Error | | p* | |
| M₀ |  | (Intercept) |  | 3.818 |  | -0.023 |  | 1.768 |  | 0.034 |  |
| M₁ |  | (Intercept) |  | 2.001 |  | -0.129 |  | 2.097 |  | 0.406 |  |
|  |  | delta TWSTRS 3 |  | 0.978 |  | -0.010 |  | 0.663 |  | 0.072 |  |
|  | | | | | | | | | | | |
| *Note.*  Bootstrapping based on 5000 replicates. | | | | | | | | | | | |
| *Note.*  Coefficient estimate is based on the median of the bootstrap distribution. | | | | | | | | | | | |
| * Bias corrected accelerated. | | | | | | | | | | | |

**Suppl. Table 1.** Bootstrapped linear regression model with 5000 replicates to examine the influence of change in pain (TWSTRS part III) on depression (BDI-II)
