# Supplementary material for: Pridinol treatment in cervical dystonia: improvement of non-motor symptoms
Source: Ann Med. 2026 Aug 20;58(1):2715209. doi: 10.1080/07853890.2026.2715209 (PMC13501772; doi:10.1080/07853890.2026.2715209)
Supplement: Supplementary file 2 [file IANN_A_2715209_SM7048.docx]

**Supplementary Figures**

**Supplementary Figure 1:** Flow diagram according to the CONSORS-statements

**Supplementary Figure 2:** Mean values and standard error of clinical scales at visits 1-3; A) DNMSQuest did not change significantly; B) EQ-5D-5L scores did not change significantly C) TWSTRS part II scores did not change significantly.
